# Advances in extraction methods, chemical constituents, pharmacological activities, molecular targets and toxicology of volatile oil from *Acorus calamus* var. *angustatus* Besser

**DOI:** 10.3389/fphar.2022.1004529

**Published:** 2022-12-01

**Authors:** Daoming Bai, Xiaoyu Li, Shengguang Wang, Tianyi Zhang, Yumin Wei, Qingquan Wang, Weichao Dong, Jing Song, Peng Gao, Yanan Li, Shaoping Wang, Long Dai

**Affiliations:** ^1^ School of Pharmacy, Binzhou Medical University, Yantai, China; ^2^ School of Pharmacy, Shandong University of Traditional Chinese Medicine, Jinan, China; ^3^ Shandong Yuze Pharmaceutical Industry Technology Research Institute Co., Ltd, Dezhou, China

**Keywords:** volatile oil, extraction, chemical constituents, pharmacological activity, toxicology, Acorus calamus var. angustatus Besser

## Abstract

*Acorus calamus* var. *angustatus* Besser (ATT) is a traditional herb with a long medicinal history. The volatile oil of ATT (VOA) does possess many pharmacological activities. It can restore the vitality of the brain, nervous system and myocardial cells. It is used to treat various central system, cardiovascular and cerebrovascular diseases. It also showed antibacterial and antioxidant activity. Many studies have explored the benefits of VOA scientifically. This paper reviews the extraction methods, chemical components, pharmacological activities and toxicology of VOA. The molecular mechanism of VOA was elucidated. This paper will serve as a comprehensive resource for further carrying the VOA on improving its medicinal value and clinical use.

## Graphical Abstract

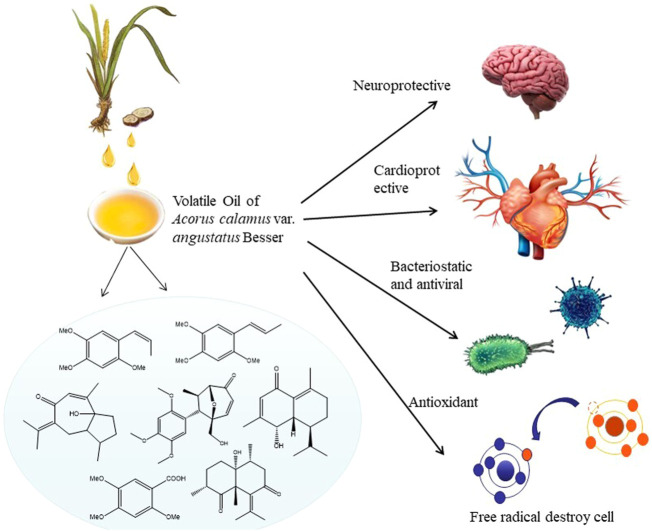

## 1 Introduction

***Acorus calamus* var. *angustatus*** Besser (ATT) is commonly known as the “sweet flag” and is native to China, India, Myanmar, Japan, Mongolia, and others (Gong et al., 2019). ATT is a grass-like, perennial herbaceous plant. It grows at an altitude of 20–2,600 m, mostly in the soils between the water and the mountains or between the water and gravel in the gully. ATT is famous for its medicinal value in Asia. It is one of the important components of the traditional systems of medicine in China and India (Zhang et al., 2015). In traditional Chinese medicine, the rhizome of ATT is often used to treat various diseases, such as anxiety, depression and other neurological diseases; abdominal tumours; epilepsy; dysentery; rheumatism, etc (Ryuk et al., 2014).

**FIGURE 1:**
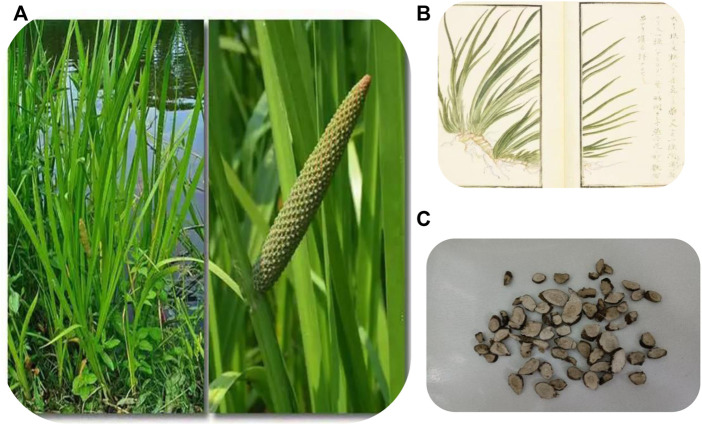
Plant morphology **(A) (B)** and section morphology **(C)** of ATT. **(A)** Is from Sohu.com, which shows the whole plant of ATT; **(B)** from *Atlas of Materia Medica,* also showing the whole plant of ATT; **(C)** is a slice of root.

Studies have shown that ATT contains volatile oil, flavonoids, alkaloids, organic acids, and other components. The volatile oil is the most important active component and has a good curative effect in treating various diseases (Ma et al., 2015). Modern medicine shows that the volatile oil components of ATT have significant beneficial effects in treating cardiovascular diseases, Alzheimer’s disease and depression. In recent years, researchers have found various volatile oil components from ATT and their beneficial effects in treating various diseases. In this paper, based on previous experimental studies, the extraction processes, chemical composition, pharmacological activity, mechanisms of action and toxicological properties of VOA were reviewed to explore its therapeutic potential and suggest future research directions. Figure 1 shows the Plant morphology and section morphology of ATT.

## 2 Technologies to extract volatile oil from *Acorus calamus* var. *angustatus* Besser

Plant volatile oils are secondary metabolites and they contain small chemical substances. The volatile oil is a complex mixture composed of dozens or hundreds of compounds. According to the chemical composition, it can be divided into terpenoid, aromatic, aliphaticand nitrogen-sulfur containing compounds. Among them, terpenoids are the main components of volatile oil (Yang, 2021). ATT has the effect of awakening spirit and benefiting intelligence, removing dampness and helping digestion, and is used in the clinic to treat various diseases. The volatile oil in ATT is the main active component. In extracting volatile oil from ATT, the technology has the greatest impact on oil quality and extraction rate. Therefore, the technology used to extract volatile oil plays a significant role in studying ATT. In the extraction of volatile oil, it is also very important to select the correct extraction procedure because improper procedures may lead to the destruction of chemical components of volatile oil, thereby affecting its biological activity. The most commonly used volatile oil extraction technologies can be divided into traditional and innovative methods. The traditional method is mainly steam distillation. It requires simple equipment, simple operational procedure and low cost. Steam distillation is also the most commonly used method for extracting volatile oil from ATT. In addition, microwave-assisted extraction is used as a method to improve efficiency. Innovative extraction methods, including ultrasonic and supercritical fluid extraction, have greatly improved the extraction efficiency and are also widely used in volatile oil production (Aziz et al., 2018). In this part, wereviews the main extraction techniques and latest developments in the technology to extract volatile oil.

### 2.1 Steam distillation method

Steam distillation (HD) is one of the most commonly used methods for extracting volatile oil from plants. It uses the principle that steam carries away volatile components. It is reported that the efficiency to extract volatile oil by HD is 93% (Aziz et al., 2018). This method prevents the decomposition of the components and reduces their boiling points, and HD is widely used due to the advantages of easy operation, low cost, high efficiency, etc (Kaya et al., 2020). However, the disadvantage of HD is the higher temperature, which may cause the thermal decomposition and hydrolysis of the compounds in volatile oil (Yang et al., 2021). In addition, emulsification may occur in this process, making it difficult to separate the oil from water. Recently, many improvements were made in the distillation equipment to overcome the challenges.

Extraction time is one of the main factors affecting volatile oil yield. Božović et al. (2017) reported the effects of distillation duration on yield and quality. Studies have shown that the time required for extraction depends on the nature of the components. Li et al. (2016) investigated the effects of soaking time, liquid-solid ratio and extraction time on the extraction process with three factors and five levels of central composite design. The extraction process was optimised using response surface methodology, and the optimum conditions are: a soaking time of 2.78 h, liquid-solid ratio of 9.09, and extraction time of 6.15 h. The average yield of volatile oil was 1.58%. Shu et al. (2018) extracted the volatile oil from ATT by HD, analysed the chemical components by GC-MS, and identified them using National Insititute of Standards and Technology (NIST) 14.0 mass spectrometry libraries. Finally, the relative contents of chemical components in different plant parts were determined by area normalisation. It is concluded that there are great differences in the volatile oil components content in different parts of ATT.

### 2.2 Supercritical fluid extraction

Supercritical fluid extraction (SFE) is a relatively new methodology for extracting volatile oil from plants. Compared with traditional extraction methods, SFE is fast, convenient, highly selective (Pourmortazavi and Hajimirsadeghi, 2007), and can be carried out at low temperatures. The solubility of the components can be fine-tuned by selecting appropriate operating conditions to exclude the extraction of unnecessary compounds (Mann et al., 2013). The most commonly used supercritical fluid is CO_2_ because of its low cost and higher safety. It reaches supercritical conditions at low pressure and temperature, and it is easy to remove from the volatile oil (Grosso et al., 2009).

In 1970, Zosel (Coelho et al., 2012) extracted caffeine from *Coffea arabica* L. by supercritical fluid extraction, the first application of this extraction technology. Since then, supercritical extraction technology has developed rapidly, and many related studies have been reported. In using supercritical technology, there are many factors affecting the extraction rate, such as sample size and bulk density, extraction time, pressure and temperature (Pourmortazavi and Hajimirsadeghi, 2007). Wang et al. (2011) established a combined technique, SFE and high-speed counter-current chromatography (HSCCC), to extract VOA from ATT at a pressure of 25 MPa and a temperature of 35°C, which has been developed and successfully applied to the extraction and separation α-asarone and β-asarone from the VOA. In addition, Wang et al. (2012) used CO_2_ SFE to extract the VOA, and optimized the SFE conditions, which were 45 MPa, 35°C, 2 h, providing a reference for the extraction of VOA.

### 2.3 Microwave-assisted water distillation extraction method

Microwave is non-ionised electromagnetic energy whose frequency ranges from 300 MHZ to 300 GHZ. The microwave energy is transmitted in waves, penetrating biological materials and entering the materials through polar molecules to generate heat (Cardoso-Ugarte et al., 2013). Microwave radiation can destroy cell walls by expanding cells, changing intracellular structure, damaging glands and oil-rich cells, accelerating the movement of aqueous solution and the diffusion of internal components. Microwave-assisted water distillation (MAHD) extraction with water as a solvent is a green and environmentally friendly method to extract volatile oil from plants (Shang et al., 2020). MAHD has been widely used in recent years as a method with short extraction time, low energy consumption, and high efficiency (Wang et al., 2021).

Studies have shown that MAHD has a higher extraction rate in a shorter time than HD (Delazar et al., 2012). The gas chromatography results showed that the concentration of oxygen-containing compounds in the essential oil (extracted using MAHD) was higher and improved the quality of volatile oil (Moradi et al., 2018). Elyemni et al. (2019) explained the difference in time between the MAHD and traditional extraction methods. In MAHD, the heat is transferred *via* radiation, conduction and convection, whereas in traditional methods, the heat is transferred through conduction and convection. Studies have shown that microwave heating creates the partial pressure gradient and causes internal overheating of the volatile compounds, resulting in faster and more effective embrittlement or rupture of cell walls (Ferhat er al., 2006; Golmakani and Rezaei, 2008; Mohammadhosseini et al., 2021). Therefore, MAHD is a new extraction technology with less time-consuming, high extraction rate, energy saving, cost reduction, and is environmentally friendly. It is a good choice for the extraction of VOA. Table 1 summarizes the advantages and disadvantages of the three extraction methods.

**TABLE 1:** Advantages and disadvantages of different extraction methods.

Method	Advantages	Disadvantages
HD	Simple operation and low cost, high extraction	Long extraction time, loss of volatile components
SFE.	Fast, convenient and highly selective	The initial investment cost of equipment is high
MAHD.	Short extraction time, low energy consumption and high extraction efficiency	Not applicable to heat-sensitive substances

## 3 Components of volatile oil from *Acorus calamus* var. *angustatus* Besser

Chemical substances such as aromatic compounds and volatile oils isolated from plants have many biological activities. Since ancient times, the roots, stems, leaves and other parts of ATT have been used to treat various diseases (Weng et al., 2019). Modern studies have shown that VOA is the main substance responsible for its medicinal value (Chen et al., 2020). It has antibacterial, anti-inflammatory, antiproliferative, hypolipidaemic, cell-protective, diuretic and antiurolith properties (Olas and Bryś, 2018). It also has a neuroprotective effect and can reduce cardiovascular and cerebrovascular complications (Lee et al., 2020). It is reported that the volatile oil in ATT can also reduce the stress-induced immunosuppression and enhance the immune function of normal rats (Khwairakpam et al., 2018). Therefore, the volatile oil components of ATT have been highly researched in China and in other parts of the world (Ma et al., 2020).

It is reported that the main bioactive aromatic components in the rhizome of ATT are α-asarone {1,2,4-trimethoxy-5-[(E)-prop-1-yl] benzene} and β-asarone {1,2,4-trimethoxy-5-[(Z)-prop-1-yl] benzene} (Das et al., 2019). The content of asarone in ATT varies with species’ types and polyploid degrees. Among them, the content in tetraploid varieties is the highest, which is 90–96% (Rajput et al., 2014). More and more volatile oil components in ATT have been found in recent years. To explore the structure-activity relationship and pharmacological activity of volatile components, chemistry of the volatile components were investigated. The commonly used analytical instruments include gas chromatography (GC) (Dubrow et al., 2022), ultraviolet spectroscopy (UV) (Lee et al., 2021), infrared spectroscopy (IR) (Zhuang et al., 2021), quadrupole time-of-flight mass spectrometry (Q-TOF) (Hawwal et al., 2021), nuclear magnetic resonance (NMR) (Schripsema et al., 2022), X-ray fluorescence spectroscopy (X-ray) (Soltanzadeh et al., 2021), and high-resolution electrospray ionization mass spectrometry (HRESIMS) (Tong et al., 2010b).

Many studies have reported volatile oil components. Dong et al. (2010b) reported 28 chemical constituents from the ethanol extract of ATT, among which a variety of volatile oil components, and three sesquiterpenes were first reported. Stahl and Keller. (1981) studied the dried rhizome of ATT and found eight new sesquiterpenes, namely, Calamusin A-Calamusin H. α-Asarone and β-asarone are the main active components of ATT possessing beneficial effects in central nervous system diseases (Shi et al., 2021). Asaraldehyde in roots and asarone in leaves are responsible for the odour of volatile oil (Du et al., 2008). Many asarone-derived phenylpropanoids were reported: (-)-R-isoacorphenylpropanoid, ent-acoraminol B, Acoraminol A, Acoraminol C (Zhang F. H. et al., 2019) etc. Bai et al. (2020) reported 25 phenylpropanoids of VOA. Many studies have reported the beneficial effects of phenylpropanoids in treating neurological diseases. Monoterpenoids are important components in VOA, and the reported monoterpenoids are α- and β-pinene, laurylene, p-cymene, terpinene-α, phellandrene-β, terpinene-γ, terpinene, decene and limonene (Lam et al., 2016a).

**FIGURE 2:**
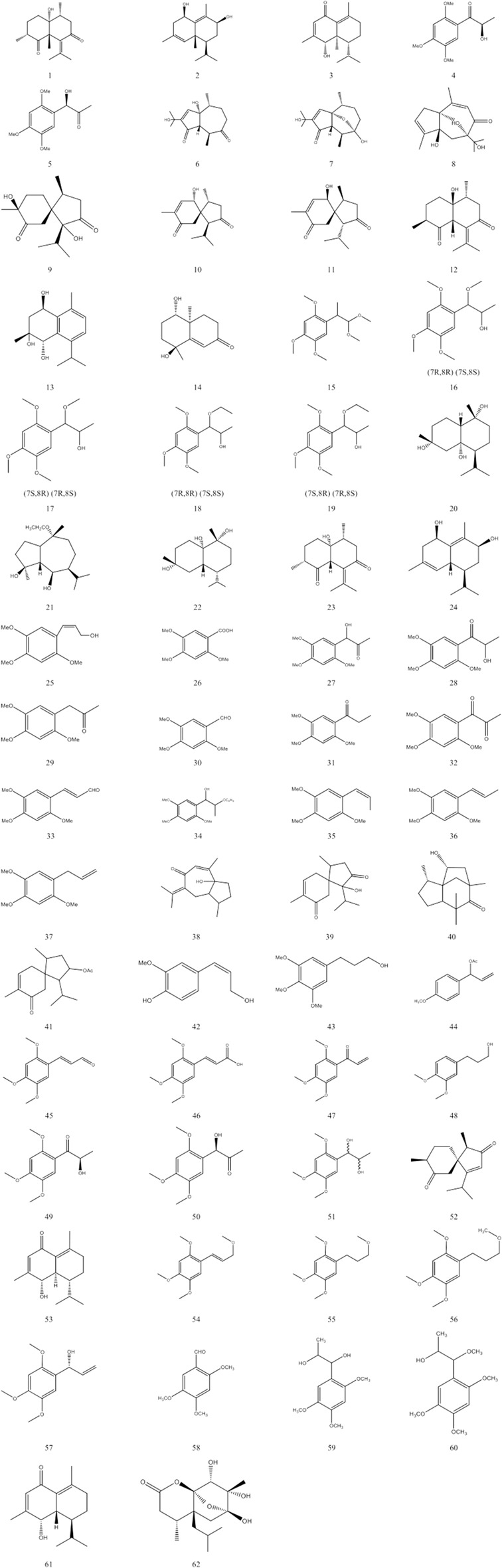
The composition of volatile of ATT and their structures.

This paper summarises the various volatile oil compounds reported in the literature and summarised in Table 2. Figure 2 shows the structures of the compounds.

**TABLE 2:** List of volatile oil components.

S.NO	Name	Extraction method	Structural characteristic	Detect method	Main findings	Refs
1	(1R,4R,6S,10R)-1-hydroxy-7 (11)-cadinen-5,8-dione (1)	MeOH extraction	C_15_H_22_O_3_, (1R, 4R, 6S, 10R), has a propan-2-ylidene group	single-crystal X-ray diffraction; UV	---	Dong et al. (2010a)
2	(2R,6R,7S,9S)-1 (10),4-cadinadiene2,9-diol (2)	MeOH extraction	C_15_H_24_O_2,_ (2R, 6R, 7S, 9S)	single-crystal X-ray diffractionUV.	---	Dong et al. (2010b)
3	tatarinowin A	were extracted with boiling H_2_O and EtOH	C_15_H_22_O_2_, (5S,6R,7R)-2-oxocadinan-1 (10), 3-dien-5-ol	HRESIMS; UV; IR; NMR	---	Tong et al. (2010a)
4	tatarinoid A	were extracted with boiling H_2_O and EtOH	C_12_H_16_O_5_, (2R)-1-(2,4,5trimethoxyphenyl) propan-2-ol-1-one	HRESIMS; UV; IR; NMR	---	Tong et al. (2010b)
5	tatarioid B	were extracted with boiling H_2_O and EtOH	C_12_H_16_O_5_, (1R)-1-(2,4,5trimethoxyphenyl) propan-1-ol-2-one	HRESIMS; UV; IR; NMR	---	Tong et al. (2010a)
6	Calamusin A	EtOH extraction,petroleum ether, EtOAc, n-BuOH purification	C_15_H_22_O_4_, the absolute configuration was determined to be 1S, 2S, 6R, 7S	NMR; IR; DEPT-135/90 spectra	---	Hao et al. (2012)
7	Calamusin B	EtOH extraction,petroleum ether, EtOAc, n-BuOH purification	C_15_H_22_O_4_, the absolute configuration was determined to be 1S, 2S, 3S, 6R, 7S		---	Hao et al. (2012)
8	calamusin C	EtOH extraction,petroleum ether, EtOAc, n-BuOH purification	C_15_H_20_O_4_, It is a racemic mixture, the configuration of a was determined to be 1R, 5S, 7S the configuration of b was determined to be 1S, 5R, 7R	NMR; IR; DEPT-135/90 spectra; ECD	---	Hao et al. (2012)
9	calamusin D	EtOH extraction,petroleum ether, EtOAc, n-BuOH purification	C_15_H_24_O_4_, the configuration of calamusin D is 1R,4S, 5S, 8S	NMR; IR; CD	---	Hao et al. (2012)
10	Calamusin E	EtOH extraction,petroleum ether, EtOAc, n-BuOH purification	C_15_H_22_O_3_	HRESIMS(m/z 273.1465 [m + Na]+); NMR; IR; UV; DEPT-135/90	---	Hao et al. (2012)
11	Calamusin F	EtOH extraction,petroleum ether, EtOAc, n-BuOH purification	C_15_H_22_O_3_, the absolute configuration of is 1R, 4S, 5R, 10R	HRESIMS (m/z 273.1465 [m + Na]+); NMR; IR; UV; DEPT-135/90	---	Hao et al. (2012)
12	calamusin G	EtOH extraction,petroleum ether, EtOAc, n-BuOH purification	C_15_H_22_O_3_, (1S, 4S, 6S, 10R)-1-hydroxy-7 (11)-cadinen-5,8-dione	HRESIMS (m/z 273.1465 [m + Na]+); NMR; IR; UV; DEPT-135/90	---	Hao et al. (2012)
13	Calamusin H	EtOH extraction,petroleum ether, EtOAc, n-BuOH purification	C_15_H_22_O_3_, the absolute configuration of Calamusin H was determined to be 2R, 4R, 5S	HRESIMS (m/z 273.1470 [m + Na]+); NMR; ECD spectra; UV; IR	---	Hao et al. (2012)
14	Calamusin I	EtOH extraction,petroleum ether, EtOAc, n-BuOH purification	C_12_H_18_O_3_, the absolute configuration of Calamusin I is 1S,4S,10S	HRESIMS (m/z 211.1324 [m + H]+); NMR; ECD spectra; UV; IR	---	Hao et al. (2012)
15	(−)-R-Isoacorphenylpropanoid/(+)-S-Isoacorphenylpropanoid	60% EtOH was extracted and purified by AB-8 macroporous resin column	C_14_H_22_O_5,_ the structures of a and b were established as (R)-1-(1,1-dimethoxypropan-2-yl)-2,4,5trimethoxybenzene and (S) 1-(1,1-dimethoxypropan-2-yl)-2,4,5-trimethoxybenzene	HRESIMS; NMR; DEPT-135; CDCl3; DMSO-d; HSQC	these compounds exhibited stronger α-glucosidase inhibitory activity than acarbose and weak AChE inhibitory activity	Dong et al. (2010a)
16	ent-acoraminol A/Acoraminol A	60% EtOH was extracted and purified by AB-8 macroporous resin column	C_13_H_20_O_5_, (7R,8R)-7-methoxy-8-hydroxydihydroasarone/(7S, 8S)-7-methoxy-8-hydroxydihydroasarone	HRESIMS; NMR; DEPT-135; CDCl3; DMSO-d; HSQC	exhibited stronger α-glucosidase inhibitory activity than acarbose and weak AChE inhibitory activity	Dong et al. (2010b)
17	ent-acoraminol B/acoraminol B	60% EtOH was extracted and purified by AB-8 macroporous resin column	C_13_H_20_O_5_, (7S,8R)-7-methoxy-8hydroxydihydroasarone/(7R,8S)-7-methoxy-8hydroxydihydroasarone	HRESIMS; NMR; DEPT-135; CDCl3; DMSO-d; HSQC	acoraminol B exhibited stronger α-glucosidase inhibitory activity than acarbose and weak AChE inhibitory activity	Dong et al. (2010a)
18	entacoraminol C/acoraminol C	60% EtOH was extracted and purified by AB-8 macroporous resin column	C_14_H_22_O_5_, (7R,8R)-7-ethoxy-8-hydroxydihydroasarone/(7S,8S)-7-ethoxy-8-hydroxydihydroasarone	Mosher’s method; HRESIMS; NMR; DEPT-135; CDCl3; DMSO-d; HSQC	exhibited stronger α-glucosidase inhibitory activity than acarbose and weak AChE inhibitory activity	Dong et al. (2010b)
19	ent-acoraminol D/acoraminol D	60% EtOH was extracted and purified by AB-8 macroporous resin column	C_14_H_22_O_5_, (7S,8R)-7-ethoxy-8-hydroxydihydroasarone/(7R,8S)-7-ethoxy-8hydroxydihydroasarone	Mosher’s method; HRESIMS; NMR; DEPT-135; CDCl3; DMSO-d; HSQC	exhibited stronger α-glucosidase inhibitory activity than acarbose and weak AChE inhibitory activity	Dong et al. (2010a)
20	1β,7α (H)-cadinane-4α,6α,10α-triol	EtOH (95%) extraction	C_15_H_28_O_3_, have a bicyclic sesquiterpene skeleton, 1β,7α (H)-cadinane-4α,6α,10α-triol	NOESY spectrum; NMR	---	(Stahl and Keller. (1981))
21	1α,5β-guaiane-10α-O-ethyl-4β,6β-diol	EtOH (95%) extraction	C_17_H_32_O_3_, 1α,5β-guaiane-10α-O-ethyl-4β,6β-diol	ESIMS; HRESIMS; NOESY	---	(Stahl and Keller. (1981))
22	6β,7β (H)-cadinane-1α,4α, 10α-triol	EtOH (95%) extraction	C_15_H_28_O_3_, 6β,7β (H)-cadinane-1α,4α,10α-triol	HSQC; HMBC; NOESY	---	(Stahl and Keller. (1981))
23	(1R,4R,6S,10R)-1-hydroxy-7 (11)-cadinen-5,8-dione	EtOH extraction	C_15_H_22_O_3_, (1R, 4R, 6S, 10R), has a propan-2-ylidene group	NMR; X-ray crystallography; HR-ESI-MS	---	Dong et al. (2010a)
24	(2R,6R,7S,9S)-1 (10),4-cadinadiene2,9-diol	EtOH extraction	C_15_H_24_O_2_, (2R, 6R, 7S, 9S)	NMR; X-ray crystallography; HR-ESI-MS	---	Dong et al. (2010b)
25	Acoramol	Ultrasonic Centrifugation of Drug Powder, Supernatant as Sample	C_12_H_16_O_4_	HPLC; ESI-QTOF-MS/MS; GC-MS; HPLC-MS; Q-TOF	---	Bai et al. (2020)
26	2,4,5-Trimethoxybenzoic acid	Ultrasonic Centrifugation of Drug Powder, Supernatant as Sample	C_10_H_12_O_5_	HPLC; ESI-QTOF-MS/MS; GC-MS; HPLC-MS; Q-TOF	---	Bai et al. (2020)
27	Tatarinoids B	Ultrasonic Centrifugation of Drug Powder, Supernatant as Sample	C_12_H_16_O_5_	HPLC; ESI-QTOF-MS/MS; GC-MS; HPLC-MS; Q-TOF	---	Bai et al. (2020)
28	Tatarinoids A	Ultrasonic Centrifugation of Drug Powder, Supernatant as Sample	C_12_H_16_O_5_	HPLC; ESI-QTOF-MS/MS; GC-MS; HPLC-MS; Q-TOF	---	Bai et al. (2020)
29	Acoramone or isoacoramone	Ultrasonic Centrifugation of Drug Powder, Supernatant as Sample	C_12_H_16_O_4_	HPLC; ESI-QTOF-MS/MS; GC-MS; HPLC-MS; Q-TOF	---	Bai et al. (2020)
30	Asaronaldehyde	Ultrasonic Centrifugation of Drug Powder, Supernatant as Sample	C_10_H_12_O_4_	HPLC; ESI-QTOF-MS/MS; GC-MS; HPLC-MS; Q-TOF	---	Bai et al. (2020)
31	Isoacoramone or acoramone	Ultrasonic Centrifugation of Drug Powder, Supernatant as Sample	C_12_H_16_O_4_	HPLC; ESI-QTOF-MS/MS; GC-MS; HPLC-MS; Q-TOF	---	Bai et al. (2020)
32	1-(2,4,5-trimethoxyphenyl) propan-1,2-dione	Ultrasonic Centrifugation of Drug Powder, Supernatant as Sample	C_12_H_14_O_5_	HPLC; ESI-QTOF-MS/MS; GC-MS; HPLC-MS; Q-TOF	---	Bai et al. (2020)
33	(E)-3-(2,4,5-Trimethoxyphenyl) acrylaldehyde	Ultrasonic Centrifugation of Drug Powder, Supernatant as Sample	C_12_H_14_O_4_	HPLC; ESI-QTOF-MS/MS; GC-MS; HPLC-MS; Q-TOF	---	Bai et al. (2020)
34	2,45-Trimethoxyl-2′-butoxy-1,2-phenyl propandiol	Ultrasonic Centrifugation of Drug Powder, Supernatant as Sample	C_16_H_26_O_5_	HPLC; ESI-QTOF-MS/MS; GC-MS; HPLC-MS; Q-TOF	---	Bai et al. (2020)
35	β-Asarone	Ultrasonic Centrifugation of Drug Powder, Supernatant as Sample	C_12_H_16_O_3_	HPLC; ESI-QTOF-MS/MS; GC-MS; HPLC-MS; Q-TOF	---	Bai et al. (2020)
36	α-Asarone	Ultrasonic Centrifugation of Drug Powder, Supernatant as Sample	C_12_H_16_O_3_	HPLC; ESI-QTOF-MS/MS; GC-MS; HPLC-MS; Q-TOF	---	Bai et al. (2020)
37	γ-Asarone	Ultrasonic Centrifugation of Drug Powder, Supernatant as Sample	C_12_H_16_O_3_	HPLC; ESI-QTOF-MS/MS; GC-MS; HPLC-MS; Q-TOF	---	Bai et al. (2020)
38	2,3,3a,7,8,8a-Hexahydro-3a-hydroxy-1,4-dimethyl-7-(1-methylethylidene)-6 (1H)-azulenone	Ultrasonic Centrifugation of Drug Powder, Supernatant as Sample	C_15_H_22_O_2_	HPLC; ESI-QTOF-MS/MS; GC-MS; HPLC-MS; Q-TOF	---	Bai et al. (2020)
39	Isocalamediol	Ultrasonic Centrifugation of Drug Powder, Supernatant as Sample	C_15_H_22_O_3_	HPLC; ESI-QTOF-MS/MS; GC-MS; HPLC-MS; Q-TOF	---	Bai et al. (2020)
40	Calamensesquiterpinenol	Ultrasonic Centrifugation of Drug Powder, Supernatant as Sample	C_15_H_24_O_2_	HPLC; ESI-QTOF-MS/MS; GC-MS; HPLC-MS; Q-TOF	---	Bai et al. (2020)
41	2-Acetoxyacorenone	Ultrasonic Centrifugation of Drug Powder, Supernatant as Sample	C_17_H_26_O_3_	HPLC; ESI-QTOF-MS/MS; GC-MS; HPLC-MS; Q-TOF	---	Bai et al. (2020)
42	(Z)-Coniferyl alcohol	Ultrasonic Centrifugation of Drug Powder, Supernatant as Sample	C_10_H_12_O_3_	HPLC; ESI-QTOF-MS/MS; GC-MS; HPLC-MS; Q-TOF	---	Bai et al. (2020)
43	3-(3,4,5-trimethoxyphenyl) propan-1-ol	Ultrasonic Centrifugation of Drug Powder, Supernatant as Sample	C_12_H_18_O_4_	HPLC; ESI-QTOF-MS/MS; GC-MS; HPLC-MS; Q-TOF	---	Bai et al. (2020)
44	1-(4-Methoxyphenyl) allyl acetate	Ultrasonic Centrifugation of Drug Powder, Supernatant as Sample	C_12_H_14_O_3_	HPLC; ESI-QTOF-MS/MS; GC-MS; HPLC-MS; Q-TOF	---	Bai et al. (2020)
45	acrylaldehyde	EtOH extraction	C_12_H_14_O_4_	ECD; NMR; HPLC; UV; IR; HPLC; MPLC; Silica gel 60 F254 plates	---	Bai et al. (2020)
46	acrylic acid	EtOAc extraction	C_12_H_14_O_4_	ECD; NMR; HPLC; UV; IR; HPLC; MPLC; Silica gel 60 F254 plates	---	Bai et al. (2020)
47	1′-oxoasarone	EtOAc extraction	C_12_H_14_O_4_	ECD; NMR; HPLC; UV; IR; HPLC; MPLC; Silica gel 60 F254 plates	---	Bai et al. (2020)
48	3-(3,4-dimethoxyphenyl) propan-1-ol	EtOAc extraction	C_11_H_16_O_3_	ECD; NMR; HPLC; UV; IR; HPLC; MPLC; Silica gel 60 F254 plates	---	Bai et al. (2020)
49	tatarinoids A	EtOAc extraction	C_12_H_16_O_5_	ECD; NMR; HPLC; UV; IR; HPLC; MPLC; Silica gel 60 F254 plates	---	Bai et al. (2020)
50	tatarinoids B	EtOAc extraction	C_12_H_16_O_5_	ECD; NMR; HPLC; UV; IR; HPLC; MPLC; Silica gel 60 F254 plates	---	Bai et al. (2020)
51	7,8-dihydroxydihydroasarone	EtOAc extraction	C_12_H_18_O_5_	ECD; NMR; HPLC; UV; IR; HPLC; MPLC; Silica gel 60 F254 plates	---	Bai et al. (2020)
52	acorusin D	EtOAc extraction	C_15_H_22_O_2_	ECD; NMR; HPLC; UV; IR; HPLC; MPLC; Silica gel 60 F254 plates	---	Bai et al. (2020)
53	tatarinowin A	EtOAc extraction	C_15_H_22_O_2_	ECD; NMR; HPLC; UV; IR; HPLC; MPLC; Silica gel 60 F254 plates	---	Bai et al. (2020)
54	(E)-1,2,4-trimethoxy-5-(3-methoxyprop-1-en-1-yl)benzene	EtOAc extraction	C_13_H_18_O_4_	ECD; NMR; HPLC; UV; IR; HPLC; MPLC; Silica gel 60 F254 plates	---	Bai et al. (2020)
55	3-(2,4,5-trimethoxyphenyl) propan-1-ol	EtOAc extraction	C_12_H_18_O_4_	ECD; NMR; HPLC; UV; IR; HPLC; MPLC; Silica gel 60 F254 plates	---	Bai et al. (2020)
56	1,2,4-trimethoxy-5-(3-methoxypropyl) benzene	EtOAc extraction	C_13_H_20_O_4_	ECD; NMR; HPLC; UV; IR; HPLC; MPLC; Silica gel 60 F254 plates	---	Bai et al. (2020)
57	γ-Asaronol	EtOAc extraction	C_12_H_16_O_4_	ECD; NMR; HPLC; UV; IR; HPLC; MPLC; Silica gel 60 F254 plates	---	Bai et al. (2020)
58	Asaronaldehyde	n-hexane extraction and purification	C_10_C_l2_O_4_	UV; ESI-MS; 1H-NMR	---	Park et al. (2011)
59	Dihydroxyasarone	n-hexane extraction and purification	C_12_H_18_O_5_	UV; ESI-MS; 1H-NMR	---	(Park et al., 2011)
60	Acoraminol B	n-hexane extraction and purification	C_13_H_20_O_5_	UV; ESI-MS; 1H-NMR	---	(Park et al., 2011)
61	3-dien-5-ol	Boiling water and EtOH extraction	C_15_H_23_O_2_	UV; IR; NMR; HR-ESIMS; EI-MS	---	Tong et al. (2010b)
62	tatarinolactone	Steam distillation; extraction and Purification of n-Butanol	C_15_H_24_O_6;_ 6,7,8-trihydroxy-4a-isobutyl-4,7-dimethylhexahydro-6,8a-epoxychromen-2 (3H)-one	UV; IR; NMR; HR-ESIMS; EI-MS	significantly inhibited SERT activity	Liang et al. (2015)

## 4 Pharmacological activities and mechanisms of volatile oil of *Acorus calamus* var. *angustatus* Besser

### 4.1 Effect on the central nervous system

#### 4.1.1 Neuroprotective effect

ATT is one of the components of Baiyou Powder in ancient China. It is a good product for benefiting vital energy. It can aromatise dampness, wake up the spleen and stomach, remove turbidity and phlegm and promote resuscitation and tranquillisation. It is an important medicine for aromaticity, tranquillisation and resuscitation. It is widely used in clinical practice for epilepsy, amnesia, stroke aphasia, Alzheimer’s disease and other diseases. Its use in these diseases indicates that ATT has the effect of tranquilising mind and brain, which is a good medicine for treating neurological diseases (Quan et al., 2017). Essential oil is the main active compound responsible for the sedative, hypnotic and anticonvulsant effects of ATT (Zhong et al., 2018). α-asarone and β-asarone are important neuroprotective components of the volatile oil (Ning et al., 2016). The quantity of these two compounds depends on the species and origin of ATT. Due to the strong lipophilicity of α-and β-asarone, their oral bioavailability is very poor. However, α-and β-asarone are widely distributed in the brain, indicating that they can penetrate the blood-brain barrier; thus, they have the potential to treat central nervous system diseases. Chellian et al. (2017) found that α-and β-asarone had a negative regulatory effect on glutamatergic neurotransmission. In an *in vitro* study, α-asarone and β-asarone inhibited the excitotoxicity induced by N-methyl-D-aspartate (NMDA) or glutamate in rat cortical preparations. In addition, Schwann cells around the peripheral axons are involved in the development, regeneration and maintenance of nerve function and structure. After β-asarone treatment, the proliferation and morphology of Schwann cells in rats were improved (Xu et al., 2016). Based on these findings, the neuroprotective effects of α- or β-asarone can be effectively used to treat neurodegenerative diseases. Reddy et al. (2015) studied the preventive effect of ATT powder on stress-induced cognitive function and the regulation of antioxidant and Na^+^-K^+^-ATPase activity. They also found that this effect may be neuroprotective against N-methyl-D-aspartic acid (NMDA) or glutamate-induced excitotoxicity.

One study explored the neuroprotective effect of α-asarone and its related mechanism against cerebral ischemia-reperfusion (CIR) stroke (Zhang et al., 2021). Its histological and flow cytometry analysis showed that α-asarone treatment reduced the damage and apoptosis of cells *in vitro* and *in vivo*. Furthermore, α-asarone decreased the expression of glial fibrillary acidic protein (GFAP), Iba-1 and LC3II/LC3I and increased the expression of p62. Moreover, α-asarone has a good neuroprotective effect, manifested in reducing epileptic seizures after stroke and improving neurological function. The results showed that α-asarone reduced CIR stroke injury by improving glial activation and autophagy.

VOA has been proven to play a neuroprotective role by protecting cells under oxidative stress (Yan et al., 2020). Oxidative stress is related to the pathogenesis of a variety of neurological diseases, excessive reactive oxygen species (ROS) production will lead to cell damage and mitochondrial dysfunction, which are key factors in the generation of neurological diseases (Witte et al., 2010; Singh and Cancelas, 2020).

Studies have found that the cytoprotective effect of VOA was related to the upregulation of peroxisome proliferator-activated receptor-γ coactivator 1-alpha (PGC-1α) expression (Chaube et al., 2015; Brandt et al., 2017), and the activation of cAMP response element binding protein (CREB) is involved in VOS-induced PGC-1α expression (Bantubungi et al., 2014; Yao et al., 2016). The specific verification process was as follows: 1) PGC-1αsiRNA knockdown resulted in decreased expression of antioxidant proteins under VOA treatment, and subsequently increased intracellular ROS level after H_2_O_2_ stress; 2) Inactivation of CREB by H89 resulted in decreased expression of PGC1α and antioxidant proteins under VOA treatment, and subsequently decreased VOA mediated cytoprotective activity after H_2_O_2_ stress. Therefore, we conclude that VOA effectively prevents H_2_O_2_ -induced cell damage by activating CREB/PGC-1α signaling in cells.

The neuroprotective effect of VOA is one of its important roles. Many studies have confirmed various molecular pathways were involved in the activity of volatile oil. More in-depth studies should be carried out to establish and confirm the neuroprotective mechanisms of ATT and its use in various diseases. The neuroprotective targets and mechanisms of ATT are summarised in Table 3.

**TABLE 3:** The neuroprotective targets and mechanisms of VOA.

Phytochemicals	Molecular mechanisms	Targets	Refs
α-asarone and β-asarone	the N-methyl-D-aspartate (NMDA) or glutamate-induced excitotoxicity in rat cortical preparations was inhibited	the excitatory amino acid carrier 1; NMDA	Chellian et al. (2017)
α-asarone and β-asarone	improved the proliferation and morphology of rat Schwann cells (RSC96)	S100 protein (a specific marker protein of Schwann) cell); neurotrophin	Chellian et al. (2017)
ATT rhizome powder	the neuroprotective effect against NMDA or glutamate-induced excitotoxicity	Antioxidants; Na-K-ATPase	Reddy et al. (2015)
α-asarone	attenuated the CIR stroke injury *via* ameliorating glial activation and autophagy	GFAP; Iba-1; LC3II/LC3I; p62	Zhang et al. (2021)
volatile oil of ATT	increased the viability of cells affected by H_2_O_2_-mediated injury, inhibited reactive oxygen species (ROS) accumulation, and induced the expression of several antioxidant proteins (SODs, GPx, and UCPs)	CREB; PGC-1α	Yan et al. (2020)

#### 4.1.2 Alzheimer’s disease

Alzheimer’s disease (AD) is an age-related neurodegenerative disease. AD is characterised by progressive memory loss and cognitive dysfunction (Dujardin et al., 2022). The accumulation of extracellular amyloid plaques and intracellular neurofibrillary tangles (NFT) formed by tau protein is considered the main pathological feature of this disease (Yang et al., 2017). Studies have shown that Chinese medicine involves a variety of mechanisms in the treatment of AD, such as inhibiting amyloid deposition and Tau phosphorylation, regulating neurotransmitters, protecting nerve cells from injury and apoptosis, and resisting oxidative stress (Soltani Khaboushan et al., 2022). ATT can improve the learning, memory and behaviour ability in AD animal models, and its main active component is asarone. Asarone is involved in many mechanisms in the treatment of AD (Mikami et al., 2021).

**FIGURE 3:**
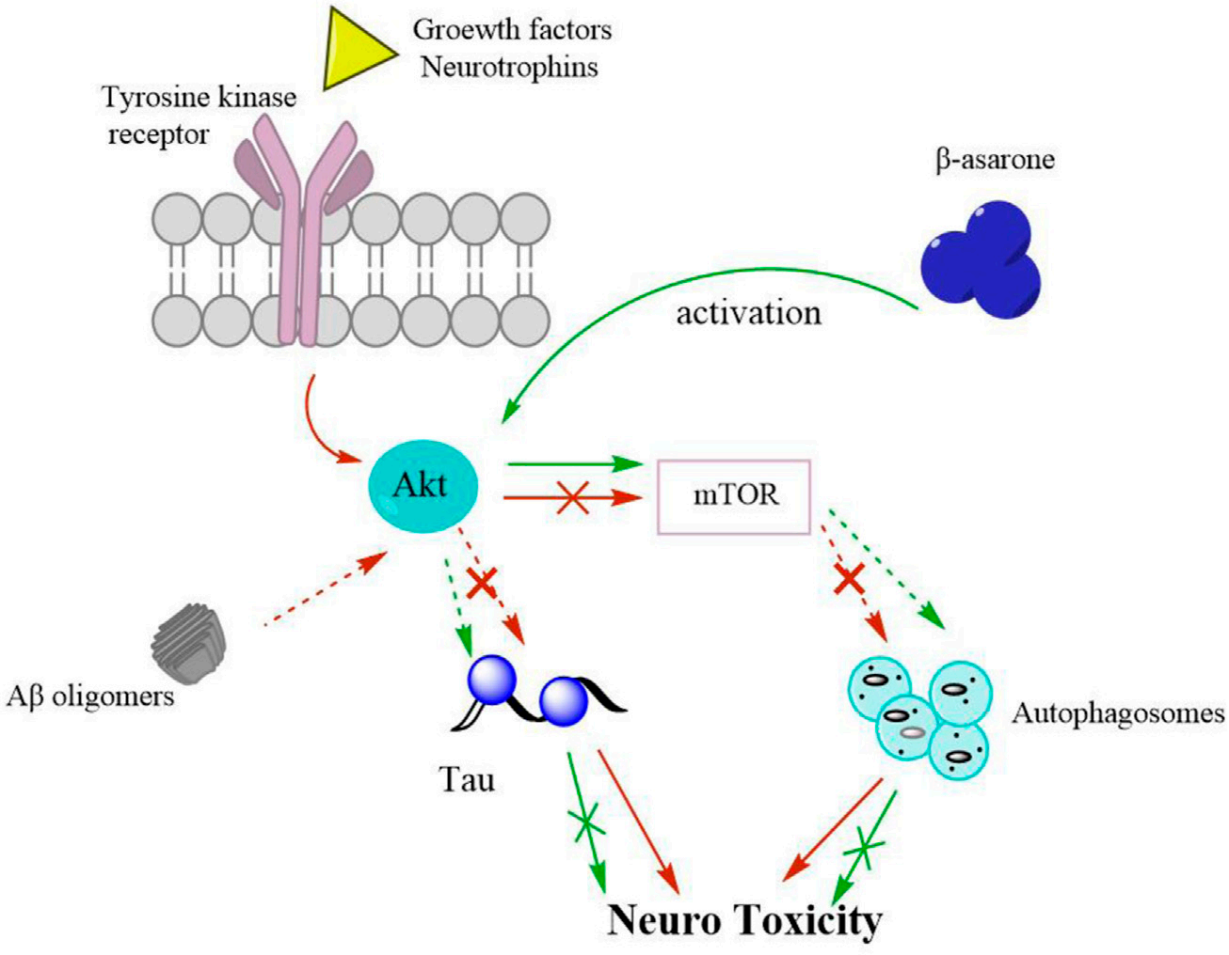
A schematic diagram shows that β-asarone protects cells from Aβ1-induced cytotoxicity by activating Akt-mTOR signaling pathway. Arrows, activation; Discontinuouslines, inhibition; Red, Aβ-induced cytotoxicity; Green, therapeutic mechanism of β-asarone on AD by activating Akt/mTOR pathway.

Recent evidence suggests that autophagy might be involved in the pathogenesis of AD (Li et al., 2021). Autophagy is a self-degradative process and plays a critical role in removing long-lived proteins and damaged organelles. A study shows that β-asarone protects cells from Aβ1-induced cytotoxicity by activating the Akt-mTOR signalling pathway, inhibits autophagy, and participates in the neuroprotective effect of β-asarone on Aβ toxicity (Figure 3). In addition, the proliferation and self-renewal of abnormal neural precursor cells (NPC) are related to AD (Mao et al., 2015). During ageing, chronic stress and central nervous system diseases, NPC proliferation and decreased self-renewal may lead to cognitive dysfunction (Lee et al., 2018). Promoting neurogenesis is considered a potential prevention and treatment strategy for AD. Studies have shown that VOA and its active component asarone can promote the proliferation of NPC.

Further analysis found that α-asarone and β-asarone are two active components of ATT in promoting neurogenesis. ATT and asarone activated extracellular signal-regulated kinase (ERK) and Akt, the two key kinase cascades in neurogenesis. Therefore, ATT and asarone can be used as potential drugs to treat age-related neurodegenerative diseases and neurodegenerative diseases.

Traditional treatment methods of AD involve targeting cholinesterase and N-methyl D-aspartate receptors. However, the aetiology of AD is complex; thus, the treatment of AD by targeting a single target is not possible. Traditional medicines such as ATT use a holistic approach and exhibit multimodal activity. Asarones and their phenylpropanoid derivatives also have protective activities against neuronal oxidative stress (Gayathri et al., 2022). Asarone also possesses antidiabetic activity by promoting insulin and insulin secretion. AD is closely associated with diabetes; thus, asarone’s antidiabetic effect may also help improve AD (Spence et al., 2017).

In addition, oxidative stress and endoplasmic reticulum stress have also been related to the pathology of neurodegenerative diseases such as AD. Oxidative stress is mainly caused by ROS. It plays an important role in cell division and mitochondrial metabolism (Venditti et al., 2013), and multiple key physiological roles such as cell differentiation (Rhee, 2006). Excessive ROS production leads to apoptosis (Schieber and Chandel 2014). Endoplasmic reticulum stress is due to misfolded protein accumulation or calcium imbalance in the endoplasmic reticulum, leading to neuronal injury or apoptosis. Therefore, drugs regulating oxidative stress or endoplasmic reticulum stress can be used as effective candidates for AD treatment. Mikami et al. (2021) found that VOA and α-asarone exert antioxidant effects in mouse macrophages (Shi et al., 2014; Lam et al., 2017). It also protected hippocampal cells from oxidative stress and endoplasmic reticulum stress by reducing ROS production and inhibiting protein kinase RNA-like ER kinase (PERK) signal transduction. It was found that VOA and α-asarone had protective effects on HT22 cell death induced by L-glutamic acid and tunicamycin. In addition, it was proved that VOA and α-asarone reduce cellular oxidative stress and endoplasmic reticulum stress by reducing the ROS production and inhibiting the PERK phosphorylation in endoplasmic reticulum stress.

VOA, together with ginsenoside, showed improved activity in improving the learning and memory ability in AD treatment; thus, there is huge potential to develop these as effective drugs for treating AD (Deng et al., 2015). The targets and mechanisms of VOA in Alzheimer’s disease are summarised in Table 4.

**TABLE 4:** The targets and mechanisms of VOA in Alzheimer’s disease.

Phytochemicals	Molecular mechanisms	Targets	Refs
β-asarone	activation of Akt-mTOR signaling pathway	Beclin-1; p-Akt; p-m TOR; neuron specific enolase (NSE)	Li et al. (2021)
asarones	promote NPC proliferation	extracellular signalregulated kinase (ERK)	Mao et al. (2015)
volatile oil of ATT	Decreased AChE activity and levels of Aβ1-42 and MDA in cortex and hippocampus; increased ChAT and SOD activities as well as BCL-2 content	acetyl cholinesterase (AChE); acetylcholine transferase (ChAT); Superoxide dismutase (SOD); malondialdehyde (MDA)	Deng et al. (2015)
			Esfandiari et al. (2018)
β-asarone	enhanced the expression of synaptophysin 1 and glutamate receptors and down-regulated aquaporin 4	synaptophysin 1; glutamate receptors; aquaporin 4	Gayathri et al. (2022)
α-asarone	improved expression of muscarinic acetylcholine receptors; attenuated the acetylcholinesterase (AchE) activity regulating locomotor hyperactivity	muscarinic acetylcholine receptors	Gayathri et al. (2022)
asarones	antidiabetic activity	promoting the secretion of insulin and incretins like GLP-1	Gayathri et al. (2022)
α-asarone	antioxidant stress	antioxidant enzyme-related genes	Mikami et al. (2021)
α-asarone	antimodulate endoplasmic reticulum stress	protein kinase RNA-like ER kinase (PERK)	Mikami et al. (2021)

#### 4.1.3 Antidepressant

Depression is a common neurological disease with a high incidence and can seriously endanger the lives of patients. In recent years, due to increased social and personal pressure, many people have depression. It is estimated that depression affects 21% of the world’s population. It is the main cause of disability and death resulting from suicide and increases the incidence of physical disorders. However, antidepressants and mood stabilisers used to treat depression, however they also induce many adverse reactions. Studies have found that plant-derived drugs are safer and effective than synthetic drugs. ATT is known to contain up to 4.86% of essential oil, mainly composed of β-asarone and α-asarone. As indicated in Table 5, the antidepressant effect of VOA has been reported in a growing number of researches. In recent years, studies have reported that VOA can inhibit glutamate-induced excitotoxicity in a concentration-dependent manner. It has neuroprotective effects on cultured cortical neurons by blocking NMDA receptor activity and has antioxidant effects *in vitro* and *in vivo*. Han et al. (2013) used a forced swimming test (FST), tail suspension test (TST) and open field test (OFT) to evaluate the antidepressant effect of essential oil and asarone. They found that injection of essential oil and asarone into mice could produce stimulation or antidepressant effects that might alter the antidepressant central nervous system, with minimal immobility. Studies (Chellian et al., 2017) have also shown that acute treatment of α-and β-asarone shows antidepressant activity in TST and FST. These results provide pharmacological support for the antidepressant effect of ATT.

**TABLE 5:** The targets and mechanisms of antidepressant effect of VOA.

Phytochemicals	Molecular mechanisms	Targets	Refs
α-asarone	through its interaction with GABAergic system	noradrenergic (α1 and α2 adrenoceptors); serotonergic (5-HT1Areceptors)	Chellian et al. (2017)
volatile oil of ATT	blocking NMDA receptor activity	NMDA receptor	Chellian et al. (2017)
β-asarone	up-regulating BDNF expression	mitogen-activated protein kinase phosphatase-1 (MKP-1); extracellular signal-regulated kinase 1/2 (ERK1/2 or mitogen-activated protein kinase)/cAMP response element binding protein (CREB)	Lam et al. (2016a)
α-asarone and β-asarone	phosphorylated CREB; enhanced neuronal differentiation induced by nerve growth factor (NGF)	the protein kinase A pathway in PC12 cells	Lam et al. (2016b)
α-asarone and β-asarone	phosphorylated CREB.	monoamine oxidase	Lam et al. (2016a)
α-asarone	improve GABA transport imbalance	---	Fee et al. (2017)
			Ghosal et al. (2017)
			Duman et al. (2019)
volatile oil of ATT	bidirectional regulatory effect on SERT activity	SERT	Zhang et al. (2019b)

In addition, the antidepressant effect of α-asarone was mediated by the interaction with norepinephrine (α1 and α2 epinephrine receptors) and serotonergic (5-HT1A receptor) systems. The serotonin transporter (SERT) is a classic antidepressant target that terminates 5-HT action on 5-HT receptors through sodium-and chlorine-dependent reuptake of presynaptic neurons by neurotransmitters (Jonathan et al., 2016). At present, most antidepressant drugs are selective SERT inhibitors (SSRI), but tianeptine as SERT enhancer has also shown antidepressant effects (Kasper and Olie, 2002), this indicates that the antidepressant effect of SERT has multiple modes of action. Recently, β-asarone ether, the main substance in the petroleum ether extract of ATT, showed significant SERT enhancement effect. Meanwhile, SERT inhibitors were also present in VOA. The coexistence of SERT enhancers/inhibitors in ATT indicated that ATT had a bidirectional regulatory effect on SERT activity (Zhang Y. et al., 2019).

It was postulated that the antidepressant effect of α-asarone might be mediated by the interaction with the gamma-aminobutyric acid energy (GABAergic) system (Chellian et al., 2016). There is some evidence that α-asarone can improve symptoms of depression caused by gamma-aminobutyric acid (GABA) dysregulation. GABA is the main inhibitory neurotransmitter system in the brain, which is responsible for the overall control and fine tuning of excitatory transmission. Most patients with depression have obvious dysregulation of GABA transport (Fee et al., 2017; Ghosal et al., 2017). The decrease of GABA in patients with depression is often accompanied by the increase of glutamic acid (Glu). This imbalance of inhibitory and excitatory transmitters is an important cause of depression (Bak et al., 2006). Glu is cleared from the synapse through the Glu transporters in astrocytes and metabolized to glutamine (Gln) by glutamine synthetase. Gln is released from astrocytes to presynaptic neurons, where it is converted into Glu through cytoplasmic glutaminase (Niciu et al., 2014). Glu is decarboxylated by glutamate decarboxylase (GAD) in neurons to form GABA. GABA is released into the synaptic gap through GABA transporters on neurons and glial cells, and activates NMDA, GABA receptors (GABARs), etc. On the postsynaptic membrane to produce inhibitory postsynaptic sites (Duman et al., 2019).

**FIGURE 4:**
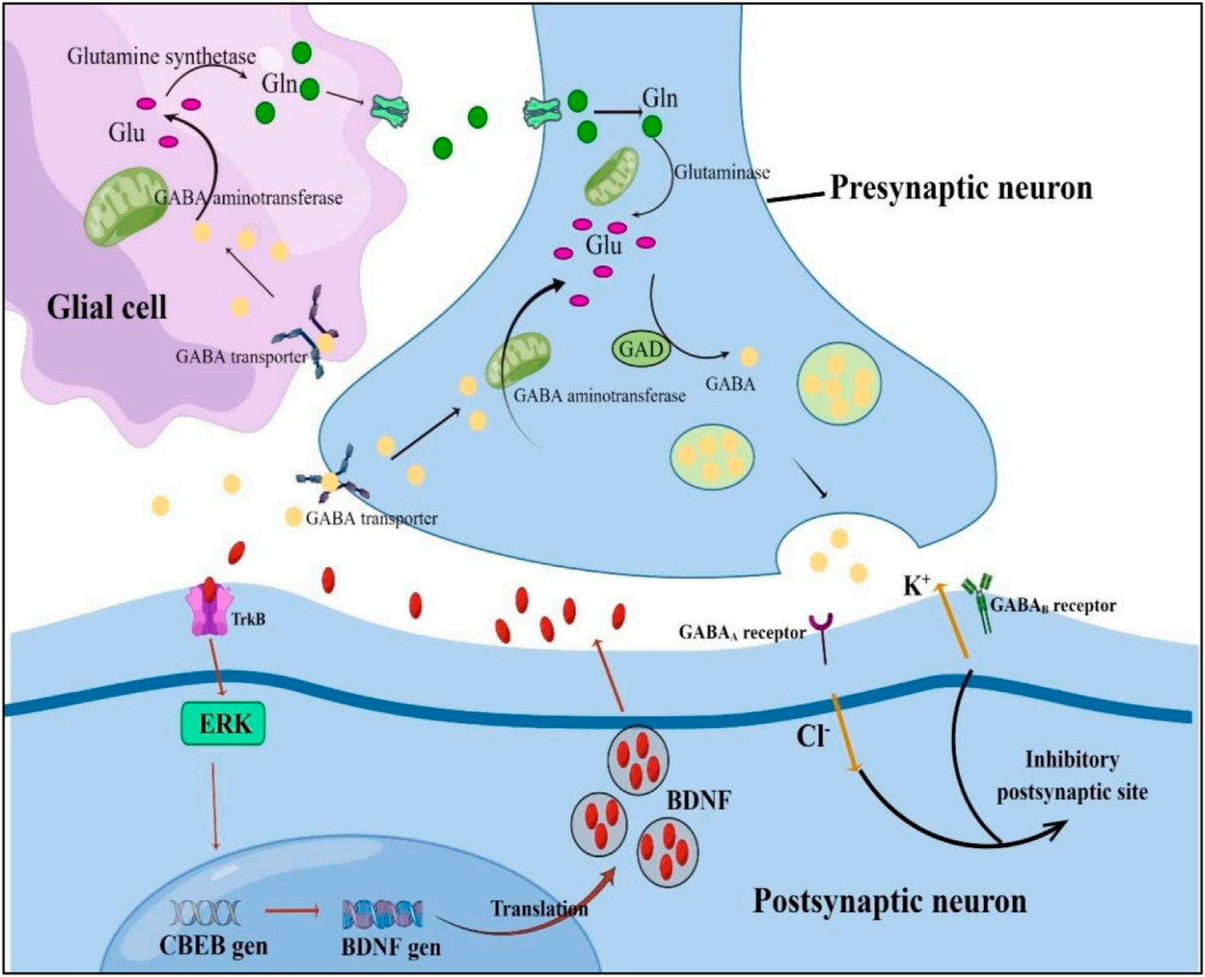
Schematic diagram of GABAergic and CREB/BDNF signaling pathways. Glu, glutamic acid; Gln, glutamine; GABA, gamma-aminobutyric acid; GAD, glutamate decarboxylase; ERK, activation; TrkB, tropomyosin receptor kinase; CBEB, cAMP response element-binding protein; BDNF, brain-derived neurotrophic factor.

On the other hand, β-asarone inhibits stress-induced hippocampal cell death (Dong et al., 2014; Sun et al., 2015), effectively reversing rats’ chronic unpredictable mild stress depression-like behaviour. In addition, β-asarone protects hippocampal neurons from chronic unpredictable mild stress-induced cell death by down-regulating mitogen-activated protein kinase phosphatase-1 (MKP-1), thereby activating extracellular signal-regulated kinase 1/2 (ERK1/2 or mitogen-activated protein kinase) or CREB signalling cascade phosphorylation, thereby up-regulating brain-derived neurotrophic factor (BDNF) expression. In addition, α-and β-asarone phosphorylated CREB by activating the protein kinase A pathway in PC12 cells (Lam et al., 2017) and enhanced neuronal differentiation induced by nerve growth factor (NGF). In another study, α-and β-asarone inhibited monoamine oxidase (MAO). Therefore, VOA and its components can provide new ideas for the development of antidepressants. Table 5 summarizes the anti depression targets and mechanisms of VOA and the And the Figure 4 shows the schematic diagram of GABAergic and CREB/BDNF signaling pathways.

#### 4.1.4 Antiepileptic

Epilepsy is a common chronic disease with prevalence rates ranging from 0.5 to 1 percent in most countries. Although there are many antiepileptic drugs, the treatment range of most antiepileptic drugs is narrow (Hazra et al., 2007). Studies have shown that ATT may inhibit symptoms of epilepsy. The electrophysiological results showed that α-asarone had a positive neuromodulation effect on GABA_A_ receptor. But this regulatory effect is not *via* inhibiting GABA uptake or GABA transaminase (Huang C et al., 2013; Bahr et al., 2019). The specific mechanism of this action is not clear (Liao et al., 1998). However, mice treated with α-asarone had a protective effect on the seizures induced by GABA_A_ receptor antagonist (pentaerythritol or indotoxin). In addition, α-asarone also has an inhibitory effect on the state of epileptic persistence in rats induced by lithium pilocarpine through the positive regulation of the GABAergic system (Chen et al., 2013). Moreover, α-asarone protected N-methyl D-aspartate (a specific NMDA receptor agonist) or guanylate or kainic acid (akainate receptor agonist) (Pages et al., 2010) induced epilepsy in mice. In addition, the radioligand binding experiment confirmed the antagonistic effect of α-asarone on NMDA receptors (Cho et al., 2002).

Further more, in the whole cell voltage clamp recording, the complement inhibition of Nav1.2 current in CNaIIA cells was observed using α-asarone treatment, whose activity is the same as sodium channel blockers (phenytoin and carbamazepine) (Wang et al., 2014). The studies have proven that α-asarone possesses a broad spectrum of antiepileptic activity against various epileptic seizures due to the activation of GABA_A_ receptors, the positive modulatory effect on Glu uptake, and antagonistic effect on Glu uptake activity at NMDA receptors and the inhibition of Nav1.2 currents. On the contrary, Zaugg et al. (2011) found that β-asarone potentiated the GABA-induced chloride current on Xenopus Oocytes expressed with rat GABA_A_ (α1β2γ2S) cDNA, using whole-cell voltage clamp recording. This finding indicates that β-asarone can promote the GABA_A_ receptor. Therefore, further studies should be conducted to confirm the detailed mechanisms of β-asarone in epileptic animal models. The specific mechanisms and targets of VOA in epilepsy are shown in Table 6.

**TABLE 6:** The targets and mechanisms of VOA in epilepsy.

Phytochemicals	Molecular mechanisms	Targets	Refs
α-asarone	positive neuromodulatory effect on GABA_A_ receptors	GABA_A_ receptors	Chellian et al. (2017)
α-asarone	Antagonistic activity against NMDA receptor	NMDA receptors	Chellian et al. (2017)
β-asarone	facilitatory effect on GABAA receptors	GABA_A_ receptors	Gayathri et al. (2022)
α-asarone	the tonic inhibition of Nav1.2 currents	Nav1.2 currents	Gayathri et al. (2022)

#### 4.1.5 Anti-anxiety

Anxiety is a response to the stress due to threats, which is normal, but overwhelming or persistent anxiety is dangerous (Peng et al., 2022). Anxiety disorders include generalised anxiety (GAD), panic disorder (PD), social anxiety disorder (SAD), and post-traumatic stress disorder (PTSD) (Crocq, 2015). This disease endangers people’s health and increases the burden on global medical and health undertakings. Many studies are underway for discovering effective drugs for the treatment of anxiety.

Studies have shown that α-asarone isolated from the volatile oil of ATT can regulate the activity of hypothalamic Fcorticotrophin-releasing factor (CR), change the central norepinephrine system, prevent the decrease of BDNF (brain-derived neurotrophic factor), tropomyosin receptor kinase (TrkB) expression in the hippocampus (Lee et al., 2014), decreased BDNF and TrkB expression in the hippocampus is the cause for the development of anxiety. In addition, the one of the effective substances α-asarone of VOA can affect the endogenous corticosterone levels in the central nervous system by regulating the hypothalamus-pituitary-adrenal axis, thereby regulating behaviour and neurochemical reactions (Lee et al., 2014). The results showed that α-asarone could significantly inhibit the increase of nutrient release factor immunoreactivity in the paraventricular nucleus cortex. The findings suggest the anxiolytic effects following the administration of α-asarone are closely associated with thalamic-pituitary-adrenal modulation in the paraventricular nucleus in the hypothalamus and activation of the thalamic-pituitary-adrenal axis (Krishnamurthy et al., 2013). In addition, tyrosine hydroxylase is also involved in stress-induced activation of the central nervous system and stress-related psychological and pathological conditions such as anxiety (Lim et al., 2012). α-asarone plays a role by stimulating the central nervous system. The therapeutic effect of anxiety disorder reverses this activity by increasing the expression of tyrosine hydroxylase in blue spots. Brain-derived neurotrophic factor (BDNF), as an important neurotrophic factor, is also involved in the aetiology and treatment of anxiety (Yi et al., 2012; Yang et al., 2004). Clinical studies have shown that stress-related mental disorders, including anxiety, are associated with decreased levels of BDNF and its receptor TrkB in the brain. Therefore, BDNF-TrkB neurotrophic signalling pathway may play a role in mediating the anti-anxiety effect of α-asarone.

Tian et al. (Tian et al., 2017) believed that the amygdala is a key brain region that coordinates negative emotional responses to threatening stimuli. Excessive excitation can promote anxiety-like behavior because of increased excitatory transmission or decreased inhibitory transmission, and its imbalance leads to the disorder of amygdala neural circuit (Wu et al., 2008). Research shows that the anti-anxiety effect of α-asarone is due to maintaining the balance of excitatory/inhibitory neurotransmitters and inhibiting the excessive excitability of pyramidal neurons in amygdala. The levels of excitatory glutamate receptors (including GluR1 and NR2A) in the amygdala of mice with anxiety symptoms were up-regulated, while the levels of inhibitory GABA_A_-α2 and GABA_A_-γ2 receptors were down-regulated. However, the upregulated levels of different NMDA receptor subtypes are also very different. Experiments have shown that blocking NR2A-containing NMDARs reduces total NMDAR currents by nearly 70%, while blocking NR2B-containing NMDARs only reduces NMDAR currents by nearly 15% (Liu et al., 2004; Zhao et al., 2005), because NMDARs containing NR2A and NR2B are associated with different intracellular cascades and play different roles in synaptic plasticity and cytotoxic potential (Wise et al., 2008; Solomon and Herman, 2009). On the other hand, NR2A/NR2B ratio plays a crucial role in synaptic function. When the NR2A/NR2B ratio increases at the protein level, it may lead to pain-related anxiety-like behavior. Inhibition of the up-regulated NR2A/NR2B ratio may be one of the mechanisms of α-asarone anti-anxiety. At the same time, BLA pyramidal neurons are thought to regulate behavior output in an adaptive way by assigning affective value to sensory cues and transmitting the signal to the efferent structure. The high excitability of these neurons can promote the activity of downstream regions, including the hippocampus, nucleus accumbens and prefrontal cortex (Rau et al., 2015), which may produce anxiety-like behavior. Experiments have shown that α-asarone has an inhibitory effect on neuronal discharge rate, which represents another possible mechanism of its anti-anxiety activity.

Chellian et al. (2017) found that α-asarone had an anti-anxiety effect in anxiety animal models such as elevated + maze (EPM), light-dark conversion, new food consumption, and marble burial tests. The commonly used anti-anxiety drugs are serotonergic (for example, buspirone is a 5-HT_1A_ partial agonist) or GABAergic (diazepam, a positive allosteric modulator of GABA_A_ receptors). In addition, the antidepressant and antiepileptic effects of α-asarone were mediated through its interaction with 5-HT1A receptors and GABAA receptors. *In vivo* studies have shown that 5-HT and GABAergic systems lack anti-anxiety activity, which should be considered in future studies. Table 7 shows the specific mechanisms and targets.

**TABLE 7:** The targets and mechanisms of VOA in anxiety.

Phytochemicals	Molecular mechanisms	Targets	Refs
α-asarone	Increasing brain BDNF.	TrkB	Lee et al. (2014)
α-asarone	affect the endogenous corticosterone levels	the hypothalamus-pituitary-adrenal axis	Lee et al. (2014)
α-asarone	inhibits the increase of nutrient release factor immunoreactivity in paraventricular nucleus cortex	the hypothalamus-pituitary-adrenal axis	Krishnamurthy et al. (2013)
α-asarone	Regulate hypothalamus-pituitary-adrenal axis	tyrosine hydroxylase in blue spots	Lim et al. (2012)
α-asarone	maintaining the balance of excitatory/inhibitory neurotransmitters	glutamate receptors (including GluR1 and NR2A); GABA_A_-α2; GABA_A_-γ2	Tian et al. (2017)
α-asarone	inhibiting the excessive excitability of pyramidal neurons in amygdala	BLA pyramidal neurons	Rau et al. (2015)

### 4.2 Cardiovascular diseases

Cardio-cerebrovascular diseases (CVDs) are global non-communicable diseases with a high mortality rate. CVDs include atherosclerosis (common coronary heart disease), cardiomyopathy, stroke, etc. CVDs pose a lot of threats to human survival. The pathogenesis of CVD is complex. It involves oxidative stress, apoptosis, myocardial energy metabolism disorders, and inflammatory response. Long-term clinical studies have found that VOA has a good curative effect on CVDs. The main compounds responsible for the cardioprotective effect are α-asarone and β-asarone. They inhibit platelet aggregation, lower blood lipid, and they can easily pass through the blood-brain barrier. In recent years, many new studies have been initiated to explore the health benefits of VOA in CVD.

**FIGURE 5:**
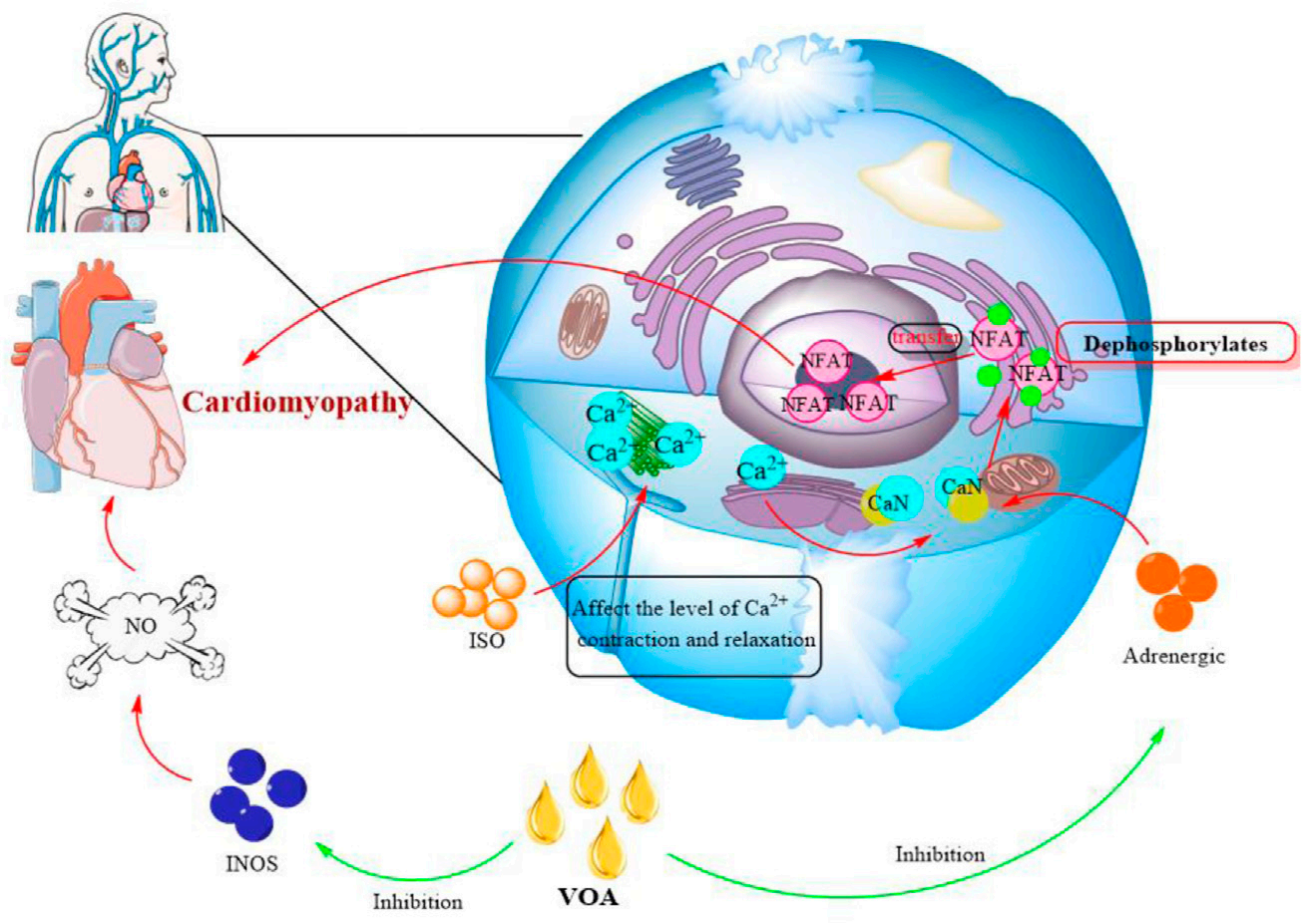
Effect of VOA on cardiomyopathy. Red, the mechanism of cardiomyopathy; Green, the effect of VOA on cardiomyopathy. INOS, NO synthase; NFAT, the nuclear factor of activated T cells; ISO, isoproterenol.

Studies have shown that VOA can alleviate isoproterenol (ISO)-induced cardiomyopathy. It is well known that can cause myocardial cell damage under pathological conditions such as hypertension, ischemic heart disease and genetic changes. ISO can affect the level of intracellular calcium contraction and relaxation and further significantly increase the activity of CaN. CaN is a serine/threonine protein phosphatase that dephosphorylates the nuclear factor (NFAT) of activated T cells and then transfers to the nucleus, leading to cardiomyopathy. The study found that taking ATT extract significantly reduced ISO-induced elevated serum CaN levels due to inhibition of adrenergic stimulation. Adrenergic stimulation is responsible for the activation of CaN, thereby limiting NFAT dephosphorylation. The increase of NO in serum is also related to myocardial injury. VOA can reduce NO levels by decreasing iNOS (NO synthase) expression (Figure 5). In addition, VOA can maintain the integrity of the cell membrane and improve calcineurin activity. This evidence shows that VOA has a cardioprotective effect on cardiomyopathy (Singh et al., 2011).

**FIGURE 6:**
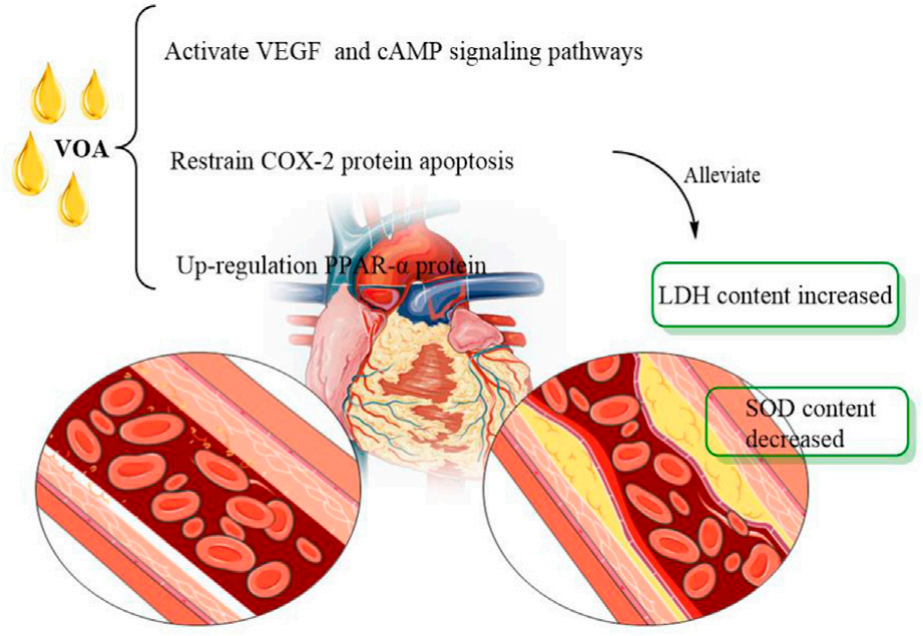
Effect of VOA on myocardial infarction. VEGF, vascular endothelial growth factors; SOD, superoxide dismutase; cAMP, also known as protein kinase A system; LDH, lactate dehydrogenase; COX-2, cyclooxygenase.

VOA also showed great potential in the treatment of myocardial infarction. Jong-Hoon Kim et al. (2014) found that VOA significantly improved the ISO-induced cardiac dysfunction. In addition, taking VOA can significantly reduce the increase of cardiac injury markers such as cardiac troponin T, tumour necrosis factor, myeloperoxidase activity and cardiac marker enzyme and prevent the consumption of antioxidant parameters. At the same time, the VOA can also inhibit the formation of malondialdehyde. Therefore, after ISO administration, the treatment of VOA can maintain the antioxidant level, inhibit the inflammatory response, and improve cardiac function and cardioprotective effect. Thus, VOA can be used as an auxiliary means for treating and preventing myocardial infarction. Another study (Zang et al., 2021) has shown that the anti-myocardial infarction effect of VOA may be attributed to the inhibition of COX-2 protein apoptosis, the up-regulation of PPAR-α protein regulating energy metabolism, and the activation of VEGF and cAMP signalling pathways. Moreover, based on network pharmacology analysis, the therapeutic effect of VOA on myocardial ischemia may be mediated by COX-2, peroxisome proliferation-activated receptor (PPAR-α), vascular endothelial growth factor (VEGF) and cyclic adenosine monophosphate (cAMP) signalling pathways. Overall, VOA attenuated isoproterenol-induced myocardial ischemia in rats, including superoxide dismutase (SOD) (Figure 6).

As one of the effective drugs for treating cardiovascular diseases, ATT has a lot of potentials for further research to understand the in-depth mechanism involved.

### 4.3 Bacteriostasis

The bacteriostatic action of VOA is one of its important pharmacological activities. Joshi (2016) evaluated the antibacterial activity of VOA and β-asarone *in vitro*. The results showed that VOA had good bactericidal activity, indicating that it could be used as a preservative. Another study (Kim et al., 2011) showed that VOA contains many methylisobutylenols with significant antibacterial activity, especially for Acnebacterium. Therefore, VOA can also be used as a cosmetic to prevent and treat *Acne vulgaris*. In addition, Lee et al. (2004) identified antifungal compounds, asarone, and cis-2,4,5-trimethoxy-1propenylbenzene. They completely inhibited the mycelial growth of some plant pathogenic fungi such as conidia, anthracnose, *Magnaporthe grisea*, and *P. multocida*. Asarones showed *in vitro* and *in vivo* antifungal activities against plant pathogens *Magnaporthe grisea* and *Bacillus rotundus*.

Ravindra et al. (2013) studied the antimicrobial activity of VOA and its main component β–asarone, the results showed that they exhibited a broad antifungal index when tested against sixteen food-infesting fungi, even at 0.25 concentration, VOA exhibited pronounced antifungal activity showing inhibition rates between 71.7% and 100% for different fungal species. At 1.0 μl/ml, β-asarone also showed 100% antifungal activity against most tested fungal species. The study also found, the aflatoxin inhibitory concentration of VOA is lower than the earlier reported VOA of *Piper betle, Ocimum gratissimum, Zanthoxylum alatum, Cicuta virosa, Cinnamomum jensenianum* (Prakash et al., 2010; Prakash et al., 2011; Tian et al., 2011; Prakash et al., 2012; Tian et al., 2012), as well as some popular preservatives (Prakash et al., 2012). Further more, VOA itself is more effective than β-asarone in terms of antimicrobial activity against food-infesting fungi and against aflatoxin secretion, reflecting the synergistic effect of some minor components in the oil on enhancing VOA activity.

Therefore, VOA can be considered as a plant-based preservative. This biologically active plant-based chemical is expected to be more advantageous than synthetic preservatives because they are easy to decompose, environmentally friendly, and mostly have no obvious residues or health risks.

### 4.4 Antioxidant activity

Many studies have shown that VOA possesses significant antioxidant effects. The neuroprotective effect of VOA is closely associated with its antioxidant activity (Muthuraman and Singh, 2011a). The beneficial effect of VOA in vincristine-induced neuropathic pain may be due to its antioxidant, anti-inflammatory, neuroprotective and calcium inhibition properties (Muthuraman and Singh, 2011b). These findings suggest the antioxidant potential of VOA, and thus, further in-depth research should be carried out. A study has reported the dual activities of VOA, anti-melanogenesis and antioxidant activity. Huang et al. (2012) studied the anti-melanogenesis and antioxidant activity of VOA for the first time. It is reported that VOA could effectively inhibit mushroom tyrosinase activity and B16F10 tyrosinase activity, reduce melanin content and exhaust intracellular reactive oxygen species. VOA effectively scavenged 2,2-diphenyl-1-picrylhydrazyl (DPPH) free radicals. VOA also showed significant reducing power and metal ion chelating activity. This finding indicated that VOA had a strong potential for antioxidant activity and inhibiting melanin. These activities suggest that VOA can be developed into skin care products.

One study has reported that the antioxidant effects of VOA are mediated *via* scavenging free radicals and increasing the antioxidant levels. The methanol extracts of ATT leaves and rhizomes have been reported by Devi and Ganjewala (2011) as a potential free radical scavenging agent. Ravindra et al. (Ravindra et al., 2013) measured the antioxidant activity of VOA and β-asarone by DPPH free radical scavenging test on TLC and by spectrophotometer according to Tepe et al. (2005). The results showed that VOA and β-asarone both exhibited dose dependent free radical scavenging activity, moreover, the antioxidant activity of VOA is believed to be related to its phytochemicals such as terpenoids and phenolics, which diffuse into the cell membrane structure and destroy it (Gholivand et al., 2010). Another study reported that VOA causes nervous protection in rats’ ischemia induced by middle cerebral artery occlusion. The neuroprotective activity is mediated *via* lipid peroxidation, glutathione level and SOD activity. Thus, VOA possesses antioxidant, neuroprotective and neurotransmitter regulatory activities, which help treat many diseases (Kardani et al., 2019).

### 4.5 Other pharmacological activities

Other pharmacological activities of VOA include protection against nickel-induced nephrotoxicity, protection against prostatic hyperplasia, protection against airway diseases, antitumour activity, wound healing activity, protection against the development of resistance to dengue virus, and antihyperglycaemic activity.

It is reported that VOA has a protective effect on nickel chloride-induced nephrotoxicity (Prasad et al., 2006). Nickel is a carcinogen and a common allergen. The nickel chloride decreases the glutathione (GSH), glutathione-S-transferase (GST), glutathione reductase (GR), lipid peroxidation (LPO), H_2_O_2_ production, blood urea nitrogen (BUN) and serum creatinine in kidneys. It also reduces the activity of glutathione peroxidase (GPx). Nickel chloride also increases the renal ornithine decarboxylase (ODC) activity and DNA synthesis in the kidney. VOA reversed the kidney’s nickel-induced changes, suggesting that VOA possesses a protective effect against NiCl_2_ nephrotoxicity in rats.

VOA can also promote wound healing. Wound healing generally includes three stages, inflammation, cell proliferation and remodelling. Ponrasu et al. (2014) reported the wound healing effect of VOA in rats. It has improved the wounds’ collagen, hexosylamine and uronic acid levels. It also increased the tensile strength of the wound by 112% and decreased the lipid peroxide levels. All these findings support the wound healing activity of VOA.

Studies have shown that VOA has strong insecticidal activity against *Zostera marina* L., *Lycoris radiata* (L'Hér.) Herb., termites, large stem borer and *Nicotiana tabacum* L. The ethanol extract of ATT has a strong repellent effect on maise weevil, S. zeamais. The supercritical fluid (CO_2_) extract of ATT has strong contact toxicity to German cockroaches and *Blattella germanica*. One study has found that the volatile oil and its components methyl eugenol, (E)-methyl isoeugenol and α-asarone from the rhizome of ATT have the potential to develop into natural fumigators or insecticides for controlling bookhoppers (Liu et al., 2013). Furthermore, Ravindra et al. (2014) studied the insect repellant, mortality, oviposition deterrent and antifeedant effects of VOA and its main component β-asarone. It was found that intact VOA was more effective than β-asarone, indicating that some additional secondary components of VOA had synergistic effects with β-asarone, resulting in enhanced activity of the intact VOA. Previous studies have found that the mode of action of VOA against insect pests is that its components have a neurotoxic effect on insects, interrupting the function of octopamine, a neuromodulator, leading to a complete collapse of the insect nervous system (Coats et al., 1991; Kostyukovsky et al., 2002). As a plant-derived compound with biodegradable, non-polluting, no significant residue or phytotoxicity, VOA shows a greater advantage in insecticide resistance than synthetic pesticides.

VOA has the function of regulating the respiratory system. It is reported that it relaxes the airways by inhibiting calcium channels and phosphodiesterase. It also contains a new anticholinergic compound that is similar to papaverine. These combinations produce a relaxing effect through a novel mechanism (Shah and Gilani, 2010). Further in-depth studies should be carried out to confirm the molecular mechanisms.

## 5 Toxicology

The main toxic components of ATT are asarones in the volatile oil. Pure α-asarone is a colourless crystalline solid, and β-asarone is a light yellow oily liquid. The major metabolite of α-asarone is (E)-30-hydroxyasarone. The epoxidation of α-asarone may lead to (E)-asarone-10,20-epoxides, which are immediately hydrolysed to form red-and threonine-configured diols (5bα5a). O-demethylation of α-asarone also occurs. (E)-asarone-10,20-epoxides are the highly active carcinogens of α-asarone (Cartus and Schrenk, 2016).

In addition, the genotoxicity of VOA is closely related to asarone epoxides. Stegmüller (2018) and colleagues found that asarone epoxides showed activity in DNA reaction tests. These asarone epoxide-related DNA adducts were also found in primary rat hepatocytes and poultry fetuses after administration of the parental compounds (Kobets et al., 2019). These evidences demonstrate that asarone-mediated carcinogenicity has a direct interaction with DNA. An acceptable daily intake (ADI) for nutritional exposure cannot be derived for β-asarone because it causes genotoxicity. Producing ATT products using diploid varieties with the lowest β-asarone content is generally recommended. ATT volatile oil and its preparations are prohibited in the United States. In the European Union, β-asarone and calamus oil is prohibited for seasoning purposes (Uebel et al., 2021).

Another study showed that β-asarone could cause duodenal cancer, inhibit the central nervous system and causes hepatotoxicity (Jaiswal et al., 2015), but the mechanism is unclear. As early as the 20th century, there were studies on the carcinogenicity of asarone. In 1967, Taylor and Jones. (1967) fed rats with different doses (500%–5,000%) of VOA containing β-asarone mixed feed for 59 weeks, in all dose groups can be seen part of the duodenal malignant tumor. Grosss et al. (1967) considered that β-asarone is carcinogenic. In 1971, the US FDA also declared that the asarone in VOA is carcinogenic (Haberman, 1971). In chronic toxicity studies, B6C3F1 male mice (preweaning, 12 days) treated with a single dose (52 mg/kg, i. p.) or continuous doses (days 1, 8, 15, and 22, with a total dose of about 1mg, i. p.) of α or β-asarone developed liver cancer at 10 months (single dose) and 13 months (continuous dose) autopsy (Wiseman et al., 1987). These studies suggest that α or β-asarone may cause hepatocellular carcinoma. In addition, α-asarone (GI50 46.0 ± 1.0 μg/ml) and β-asarone (GI50 40.0 ± 2.0 μg/ml) were cytotoxic to human hepatocytes (THLE-2) cells (Patel et al., 2015). It is worth noting that the reports of carcinogenicity and mutagenicity of VOA are concentrated in the sixties and seventies, in recent years, even reported that VOA has anticancer activity. Therefore, it seems that further *in vivo* studies are needed to confirm the hepatotoxic effects of asarone on chronic administration.

In the acute toxicity test (Han et al., 2013), intragastric administration of 400 mg/kg and 500 mg/kg asarone had toxic effects, but it was mild. The toxicity of 600 mg/kg dose was enhanced, and the mortality of mice was 33.3% (3/10) at 24 h after administration. The toxicity of 700 mg/kg dose was further enhanced, and the mortality of mice was 90% at 2 h after administration. At a dose of 800 mg/kg for 0.5 h, all mice died. The results showed that when the dose of β-asarone was higher than 500 mg/kg, it could cause acute death in mice, and the toxicity of β-asarone increased with the increase of dose. The toxic time of β-asarone mainly appeared in 15 min-6 h after administration. The main symptoms of mice after administration in this study were eyelid closure, dyskinesia, clonic and tonic convulsions, indicating that the role of β-asarone is closely related to the central nervous system, but its toxic target organs need further study. Acute toxicity test is of great significance to preliminarily clarify the toxicity of drugs and understand their possible toxic target organs, moreover, the information obtained from single dose toxicity test has important reference value for the initial dose design of repeated dose toxicity test. However, the acute toxicity test schedule is short, and the pathological changes of tissues and organs require a certain time process. Therefore, if the toxic effect of the drug is to be further determined, the toxicity test of repeated administration is required.

Shang (2015) found that α-asarone can lead to down-regulation of selenoprotein (Sepn1) gene expression, thereby opening the blood-brain barrier, its brain activity can help us solve the problem of drug delivery across the blood-brain barrier. However, α-asarone shows reproductive toxicity to zebrafish embryo development, and its biosafety is worrying. Salazar et al. (1992) administered different doses of α-asarone to pregnant mice, the results showed that the 60 mg/kg dose group observed obvious maternal toxicity, decreased weight gain, and multiple fetal malformations, including hydrocephalus, multiple ribs, deformed feet and cleft lip. Chamorro et al. (1998) administered male mice with α-asarone at doses of 0, 10, 30 mg/kg per day for 5 days, and found that the failure rate of sperm implantation increased, and sperm aggregation and activity decreased, suggesting that α-asarone had a direct toxic effect on sperm.

In addition, β-asarone can dose-dependently inhibit the frequency, amplitude, duration time, area, and significantly inhibit excitatory postsynaptic currents of tonic/expiratory airway preganglionic parasympathetic motoneurons/inspiratory airway preganglionic parasympathetic motoneurons with significantly reduction on frequency and amplitude (Qiu et al., 2011), suggesting β-asarone may bechance the inhibition of neurotransmission in the medullary respiratory neural network that leads to acute respiratory disorder.

The toxicity of VOA also shows its tropism to insects (Chen et al., 2015). Yooboon et al. (2019) studied the cytotoxicity of β-asarone on SF9 insect cells. They found that the morphological changes of cells treated with β-asarone were typical apoptosis, including adhesion loss, cell shrinkage, and small apoptotic bodies. Moreover, the DNA ladder structure in the analysis of SF9 cells and Annexin V treated with β-asarone confirmed that this compound could induce insect cell apoptosis. These results suggest that apoptosis induction is one of the mechanisms of β-asarone involved in inhibiting insect cell proliferation. Therefore, the use of ATT should be strictly controlled dose, so as not to cause toxic reaction, harm people’s health.

In traditional medicine, ATT is often used in combination with ginseng, tuckahoe, bupleurum, polygala and other medicinal materials (Cai, et al., 2021; Wu, et al., 2022). The relevant long-toxic acute toxicity test (Zhang, et al., 2013; Zhou, et al., 2018) and clinical results (Bao, et al., 2011) show that the compatibility of drugs can effectively reduce the toxic reaction caused by ATT, which is considered to be the result of the interaction of drugs during use. However, reports on the mechanism of attenuation are almost blank, which will be one of the focuses of future research.

## 6 Summary

ATT is a famous medicinal plant, which rhizomes are widely used in China, India, the United States, Thailand and South Korea, and is often used to treat neurological diseases, cardiovascular and cerebrovascular diseases, etc. ATT contains volatile oil, organic acids, flavonoids and other active components, among which volatile oil is the main component of various pharmacological effects of ATT. This review introduces several commonly used extraction methods of volatile oil, including steam distillation, supercritical fluid extraction and microwave-assisted extraction. Among them, steam distillation is the most classical method of volatile oil extraction. It is easy to operate, has a high extraction rate, and often becomes the first choice for volatile oil extraction. However, steam distillation is time-consuming and destroys the thermally sensitive components. Therefore, supercritical extraction and microwave-assisted extraction are favoured under the condition of allowable cost as they are fast, convenient, and highly selective. This paper summarises the compounds present in the volatile oil. In recent years, various modern instruments have been used to isolate the compounds from volatile oil. Their structure-activity relationships have been explored. The ultraviolet, infrared spectroscopy, gas chromatography, etc. are the main analytical techniques used to isolate compounds from VOA.

In addition, the pharmacological activities of ATT volatile oil for its activity on the central nervous system, cardiovascular and cerebrovascular systems, antibacterial and antioxidant activities were summarized in this review, some molecular mechanisms of action and molecular pathways of VOA were also mentioned. Asarone is the major bioactive compound in the volatile oil of ATT, responsible for many pharmacological activities, and it is an effective substance that can bring enlightenment to the future development of new drugs. However, in-depth studies should be carried out to explore further the mechanisms involved in the various pharmacological effects of VOA, to provide sufficient scientific basis and new insights for future quality assessment, clinical application and drug discovery.

However, it should be noted that VOA has cardiotoxicity, hepatotoxicity, reproductive toxicity and carcinogenicity. Tetraploids of ATT are highly toxic and were banned as drugs and condiments in many countries. Diploids are less toxic; thus, it is preferred in medicine and other industries. The asarone in VOA is found to be principally responsible for the toxic effects of VOA. For better application of VOA, safety assessment should be given priority. In China, VOA has been clinically tested to treat respiratory and nervous system diseases. Therefore, the toxicity of VOA should be observed and evaluated in combination with clinical experience, such as dose, drug use time, syndrome and target system, so as to avoid harm to human body. In addition, many components in VOA can be well extracted by ethanol, so the development or monitoring of ATT preparations using ethanol solvents also requires vigilance. ATT is often used in combination with other herbs in clinical applications, but there are few reports on their interactions. In the future, we can focus on the synergistic or antagonistic effects of some herbs or drugs with ATT. Finally, the study found that the content of ATT substances from different sources is very different. Therefore, supervision should be strengthened to identify ATT authoritatively before clinical application, so as to achieve better quality control.

As the main active ingredient of ATT, VOA has very high medicinal value, and is worthy of further study to develop better new drugs. At the same time, it is also being used as a condiment and natural insecticide. Thus, VOA has multifaceted potential in medicine and agriculture, which requires further in-depth studies. It is hoped that this review will stimulate interest in the ethnomedicine community and lead some efforts to modernize medicinal plants and learn from each other‘s strengths to make VOA better serve humans.
